# Exploring the Distribution of Genetic Markers of Pharmacogenomics Relevance in Brazilian and Mexican Populations

**DOI:** 10.1371/journal.pone.0112640

**Published:** 2014-11-24

**Authors:** Vania Bonifaz-Peña, Alejandra V. Contreras, Claudio Jose Struchiner, Rosimeire A. Roela, Tatiane K. Furuya-Mazzotti, Roger Chammas, Claudia Rangel-Escareño, Laura Uribe-Figueroa, María José Gómez-Vázquez, Howard L. McLeod, Alfredo Hidalgo-Miranda, Esteban J. Parra, Juan Carlos Fernández-López, Guilherme Suarez-Kurtz

**Affiliations:** 1 Computational Genomics Consortium, National Institute of Genomic Medicine, Mexico City, Mexico; 2 Nutrigenetics and Nutrigenomics Laboratory, National Institute of Genomic Medicine, Mexico City, Mexico; 3 Programa de Computação Científica, Fundação Oswaldo Cruz, Rio de Janeiro, Rio de Janeiro, Brazil; 4 Departamento de Radiologia e Oncologia, Universidade de São Paulo, São Paulo, São Paulo, Brazil; 5 Genotyping and Expression Analysis Unit, National Institute of Genomic Medicine, Mexico City, Mexico; 6 DeBartolo Family Personalized Medicine Institute, University of South Florida Moffitt Cancer Center, Tampa, Florida, United States of America; 7 Cancer Genomics Laboratory, National Institute of Genomic Medicine, Mexico City, Mexico; 8 Department of Anthropology, University of Toronto at Mississauga, Mississauga, Ontario, Canada; 9 Divisão de Farmacologia, Instituto Nacional de Câncer, Rio de Janeiro, Rio de Janeiro, Brazil; 10 Institute of Mathematical Sciences, Claremont Graduate University, Claremont, California, United States of America; 11 Pharmacogenomics Department, Pharmacogenetics for Every Nation Initiative, Tampa, Florida, United States of America; University of Florida, United States of America

## Abstract

Studies of pharmacogenomics-related traits are increasingly being performed to identify loci that affect either drug response or susceptibility to adverse drug reactions. However, the effect of the polymorphisms can differ in magnitude or be absent depending on the population being assessed. We used the Affymetrix Drug Metabolizing Enzymes and Transporters (DMET) Plus array to characterize the distribution of polymorphisms of pharmacogenetics and pharmacogenomics (PGx) relevance in two samples from the most populous Latin American countries, Brazil and Mexico. The sample from Brazil included 268 individuals from the southeastern state of Rio de Janeiro, and was stratified into census categories. The sample from Mexico comprised 45 Native American Zapotecas and 224 self-identified Mestizo individuals from 5 states located in geographically distant regions in Mexico. We evaluated the admixture proportions in the Brazilian and Mexican samples using a panel of Ancestry Informative Markers extracted from the DMET array, which was validated with genome-wide data. A substantial variation in ancestral proportions across census categories in Brazil, and geographic regions in Mexico was identified. We evaluated the extent of genetic differentiation (measured as *F_ST_* values) of the genetic markers of the DMET Plus array between the relevant parental populations. Although the average levels of genetic differentiation are low, there is a long tail of markers showing large frequency differences, including markers located in genes belonging to the Cytochrome P450, Solute Carrier (SLC) and UDP-glucuronyltransferase (UGT) families as well as other genes of PGx relevance such as *ABCC8, ADH1A, CHST3, PON1, PPARD, PPARG*, and *VKORC1*. We show how differences in admixture history may have an important impact in the distribution of allele and genotype frequencies at the population level.

## Introduction

The development of high-throughput and rapid genotyping technologies in parallel with the completion of the Human Genome Project led to a wealth of information on human genetic diversity and its impact on disease susceptibility and drug response. Pharmacogenetics and pharmacogenomics research explores the contribution of genetic individuality to variability in drug response, to provide relevant information for personalized drug therapy. A common finding in PGx studies is that the frequency of genetic variants associated with drug response differs across and within populations. One distinct example is the *VKORC1* 3673G*>A* transition, a major determinant of warfarin dose requirement for appropriate anticoagulation [1]. The frequency of the *3673A* allele, associated with low warfarin dose, ranges from <10% in sub-Saharan Africans to>90% in Southeast Asian populations [2], and from 10 to 40% among the predominant population strata of Brazil [3]. This variability has a major impact on the usefulness of the *VKORC1* 3673G*>A* SNP to predict warfarin dosage across populations worldwide [1], [4], [5]. A corollary to human diversity is that differences in frequency of clinically-relevant genetic variants might be used advantageously for PGx-informed drug therapy. Either to prevent adverse effects (e.g. carbamazepine-induced Stevens-Johnson syndrome in Southeast Asian population) to maximize clinical benefit or to rescue withdrawn medicines for use in populations which might not be adversely affected. The Pharmacogenetics for Every Nation Initiative (PGENI) addresses these goals, through the creation of an international consortium to assess the prevalence of genetic variants related to pharmacological response in an ethnically diverse set of samples, coming from different participating countries [6]. These include Brazil and Mexico, the two most populous countries in Latin America, with 194 million [7] and 112 million [8] people, respectively, which are the focus of the present study. Admixture of Native American, European and sub-Saharan African ancestral roots is extensive in both countries, but their population structure is quite different: European and sub-Saharan ancestry predominate largely over Native American ancestry in Brazilians [9], [10] whereas in Mexico the main ancestral contribution is European and Native American, and the African contribution is relatively small [11], [12], [13]. Together, Brazilian and Mexican populations provide an interesting illustration of the heterogeneity of Latin American peoples, regarding the kaleidoscopic combinations of individual proportions of Native American, European and sub-Saharan African ancestries.

In this study, we used the Affymetrix Drug Metabolizing Enzymes and Transporters (DMET) Plus array to characterize the distribution of PGx polymorphisms in a combined cohort of Brazilians and Mexicans. The DMET array interrogates variants in 231 genes involved in drug pharmacokinetics [14]. We evaluated the admixture proportions in the Brazilian and Mexican samples using a panel of Ancestry Informative Markers extracted from the DMET chip, which was validated with genome-wide data. We discuss the implications of the history of admixture in Brazil and Mexico for the distribution of genetic variants of PGx relevance.

## Materials and Methods

### Study participants and sample collection

The Brazilian cohort consisted of 268 healthy, unrelated adults recruited in the city of Rio de Janeiro in the Southeast region. The study protocol was approved by the Ethics Committee of the Instituto Nacional de Câncer, Rio de Janeiro. Each individual signed an informed consent to participate, and was asked to self-identify according to the “race/color” classification scheme adopted by the official Brazilian Census [7]. The cohort comprised 89 individuals in the branco (White), 90 individuals in the pardo (Brown) and 89 individuals in the preto (Black) Census categories. These “Color” categories will be capitalized to call attention to their special meaning in the context of the Brazilian census classification.

The Mexican cohort included 45 Native American Zapotecas (NAT) individuals and 224 self-identified Mestizo individuals (MEX) from 5 states located in geographically distant regions in Mexico. In our study, the Native American parental frequencies were estimated based on a Zapoteca sample recruited in the State of Oaxaca. Previous research has indicated that Zapotecas were the most useful population for building an admixture mapping map for Latino populations [11], [13], [15]. However, it is important point out there is not a single native group that could represent the full ancestry of Native American component in the Americas. Unfortunately, the Zapoteca sample was the only Native American sample that was available to us, and we could not evaluate the extent to which the allele frequencies observed for the panel of AIMs selected from the DMET array may differ between the Zapotecas and other Native American groups throughout the Americas. The Mestizos were recruited in the States of Sonora in the north, Guerrero in the south-Pacific, Guanajuato in the center, Yucatan in the southeast and Veracruz in the center-Gulf. The protocol was approved as part of a comprehensive genotypic characterization within the Mexican Genome Diversity Project (MGDP) [11] by the Scientific, Ethics, and Biosafety Review Boards of the National Institute of Genomic Medicine, where all Mexican participants signed an informed consent in their native language.

### Genotyping with the DMET Plus platform

Samples were genotyped using the Affymetrix DMET Plus platform, using standard protocols. This platform interrogates 1,936 genetic variants across 231 genes of PGx relevance, including biallelic and triallelic SNPs, copy-number variants and insertion/deletions. Quality control tests were performed on data using PLINK [16]. We excluded individuals with more than 5% missing genotypes and included only SNPs with at least 95% genotyping rate (<5% missing). The final dataset was based on 1,647 genetic markers in 214 genes and 537 individuals that comprise Brazilians, and Mexican Native Americans and Mestizos populations. Data are provided as Data S1 (Supporting Information - Compressed/ZIP File Archive: Supporting_information_file-1-genotyping_data.zip).

### Estimation and validation of ancestry proportions

In order to evaluate the ancestry proportions of the Brazilian and Mexican samples, we identified 71 unlinked Ancestry Informative Markers (AIMs) from the DMET Plus array. The selection of AIMs was based on frequency differences between the three main parental groups relevant for Latin American populations: European, African and Native American. We used as representatives of the parental groups 59 individuals from Europe (Hapmap CEU from northwestern Europe), 208 individuals from Africa (Hapmap YRI from Ibadan, Nigeria combined with Hapmap LWK from Webuye in Kenia) [17], and 45 Native Americans (Zapotecas from the State of Oaxaca, Mexico).

To validate the DMET Latin American AIMs panel, we used the program STRUCTURE v 2.3.4 [18], [19], [20] to estimate, based on the DMET AIMs panel, the individual ancestry proportions of the 224 Mexican Mestizo individuals analyzed in this project. The estimates obtained were then compared with genome-wide estimates (based on the Affymetrix 100K and Illumina 550K arrays) available for a subset of the samples [11], [21], [22].

### Principal Component Analysis and estimates

A Principal Component Analysis (PCA) was carried out with the program EIGENSOFT [23]. Analysis based on genotype data for the Brazilian and Mexican admixed samples, as well as representatives of the three parental populations: HapMap Europeans *CEU* (EUR), Africans *YRI+LWK* (AFR) and Native Americans (NAT). The program EIGENSOFT was also used to obtain *F_ST_* estimates for all the pairwise comparisons.

### Genetic differentiation analysis

The program PHASE v 2.1.1 [24], [25], [26] was used to estimate haplotype frequencies in the genes included in the DMET Plus microarray. Allele frequencies were estimated with the toolset PLINK [16]. This program was also used to perform chi-square tests evaluating allele differences between populations. The p-values were adjusted for multiple testing using Bonferroni's correction. We applied Wright's *F_ST_* to allele frequencies to characterize differentiation at specific SNPs as previously reported [27].

### Graphical representation of the log odds of having a variant of PGx relevance depending on admixture proportions

We assessed the relationship between allele frequencies and ancestry proportions by fitting non-linear piece-wise smooth logistic regression models [28]. In these models, the response variable was the presence/absence of the relevant PGx variant, and ancestry was entered in the model as a predictor variable transformed as a linear tail-restricted cubic spline allowing for nonlinear contributions. The result of the model fitting exercise is presented as surface plots describing the predicted relationship between the frequency of the relevant variant, expressed as the natural log odds [i.e. ln(p/(1–p), where p is the proportion of variant alleles] and ancestry. This method, described in Harrell [28] is implemented as function ‘lrm’ available in the R package ‘rms’ [29]. Analysis of variance tables describe the Wald statistics for testing the model components.

## Results

### Estimation and validation of ancestry proportions

We estimated ancestry proportions in the Brazilian and Mexican samples using a panel of 71 unlinked, highly informative AIMs included in the DMET Plus array. The panel was identified based on the frequency differences between the relevant parental populations (African, European and Native American). Detailed information about the panel of AIMs, including parental frequencies, is provided in Table S1. The program STRUCTURE was used to estimate the individual admixture proportions, as well as the average admixture proportions in each sample.

Table 1 shows the average African, European and Native American genetic contributions to the Brazilian sample stratified by census categories, and to the Mexican sample stratified by State. The average African, European and Native American contributions to the Brazilian sample were estimated to be 29%, 62% and 7% respectively. The African contribution is highest for Black Brazilians (54% vs. 25.3% in Brown and 9.7% in White individuals). Conversely, the European contribution is highest for the White category (84.6% vs. 64.7% in Brown and 38% in Black individuals). The average Native American ancestral contributions range between 5.6% and 10% across the 3 Color groups. In contrast to Brazil, where the major ancestral contributions are from Europe and Africa, in Mexico the predominant ancestral contributions are Native American and European (55.9% and 38.8%, respectively), with a relatively small contribution from Africa (5.3%). There is evidence of geographic variation in ancestral contributions in Mexico, with higher European admixture in the Mestizo sample from the State of Sonora in the North (70% vs. 18% in Guerrero) and higher Native American contributions in the state of Guerrero (74% vs. 26% in Sonora). We observed a substantial variation of individual admixture proportions in the samples of Mexico and Brazil, as depicted in Figure S1, which shows the individual admixture estimates using a bar plot.

**Table 1:** Ancestry proportions of Mexican and Brazilian populations.

Population	EUR	AFR	NAT
EUR	0.966	0.015	0.018
AFR	0.029	0.954	0.017
NAT	0.022	0.014	0.964
BZ.BK	0.381	0.536	0.083
BZ.BN	0.647	0.253	0.1
BZ.WT	0.846	0.097	0.056
GUA (MEX)	0.396	0.058	0.546
GUE (MEX)	0.184	0.072	0.744
SON (MEX)	0.701	0.043	0.256
VER (MEX)	0.281	0.045	0.674
YUC (MEX)	0.378	0.048	0.574

Ancestry proportions were calculated using Structure software (unsupervised clustering model) with a panel of 71 ancestry informative markers of 3 ancestral populations. CEU (EUR), YRI-LWK (AFR), Zapoteca (NAT). As well as other sub-populations, from Brazil we have Brazilian Black (BZ.BK), Brazilian Brown (BZ.BN), Brazilian White (BZ.WT), and five Mexican Mestizo: Guanajuato (GUA) geographically located in the center of the country; Guerrero (GUE) in the south-Pacific; Sonora (SON) in the north; Veracruz (VER) in the center-Gulf and Yucatan (YUC) in the southeast.

In order to evaluate the discriminatory power of the AIMs panel, we estimated the correlation of the individual admixture estimates obtained with the panel of 71 DMET AIMs with estimates based on genome-wide data for Mexican Mestizo individuals for which data were available (74 individuals genotyped with the Illumina 550K array and 68 genotyped with the Affymetrix 100K array, Figure S2). We observed very high *R^2^* values for the major ancestral components of the Mexican Mestizo population (Illumina 550K: 0.92 for Native American and 0.91 for European; Affymetrix 100K: 0.91 for Native American and 0.89 for European). The *R^2^* value observed for the African ancestral contribution was lower (Illumina 550K: 0.42 and Affymetrix 100K: 0.32), as expected given the relatively small average African ancestry observed in the Mexican Mestizo sample.

**Figure 1:**
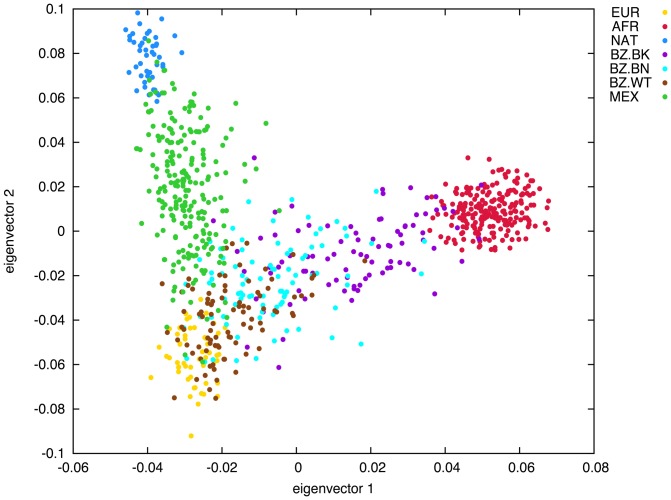
Principal Component (PC) analysis. The first two PCs were plotted. The plots are based on data for 1,647 SNPs available for two HapMap samples (EUR, AFR), Black, Brown, White Brazilians (BZ.BK, BZ.BN, BZ.WT) and Mexican Natives and Mestizos (NAT, MEX).

A principal component analysis (PCA) was performed to describe the diversity of Mexican and Brazilian populations in relation to the three ancestral populations (Figure 1). The PCA plot highlights the differences in admixture history between the Brazilian and Mexican samples, in agreement with the admixture results observed with the program STRUCTURE. The samples from Brazil are widely distributed between the samples from Europe and Africa. In contrast, Mexican Mestizos are located between the Native American and European samples in the plots.

### Genetic differentiation and haplotype diversity for DMET Plus markers

We calculated the *F_ST_* statistic, which is a measure of the degree of genetic differentiation between populations, for all the markers included in the DMET Plus array. *F_ST_* was calculated for all possible pairwise population combinations of the parental samples (African-European, African-Native American and European-Native American). Table S2 shows the average and minimum-maximum range of *F_ST_* values for all the pairwise comparisons. Figure S3 shows the distribution of *F_ST_* values for all the pairwise comparisons in a graphical format. The average *F_ST_* values are low, indicating reduced genetic differentiation. However, it is important to note the broad distribution of *F_ST_* values. While most of the genetic markers have low genetic differentiation, there are many outliers with high *F_ST_* values. The highest average *F_ST_* values are observed between the African and the Native American samples (*F_ST_* = 0.085). The *F_ST_* values observed for the African-European and European-Native American comparisons were 0.059 and 0.062, respectively. The average genetic differentiation between the Brazilian and Mexican samples with respect to the parental samples is lower than the genetic differentiation observed between the parental samples (*F_ST_* = 0.012), as expected given the history of admixture in Brazil and Mexico.

We also explored haplotype diversity using the program PHASE. We used this program to estimate haplotype frequencies based on 1,647 biallelic SNPs located on 214 DMET genes. We restricted our analysis to common haplotypes with frequencies equal or higher than 5% in each sample. We identified slightly more than 1,000 different haplotypes with these characteristics. The African sample shows the highest haplotype diversity (more than 68% of the common haplotypes are found in the African sample) and the Native American sample shows the lowest haplotype diversity (only 46.5% of the common haplotypes are found in the Native American sample). In agreement with expectations based on population history, the Brazilian sample has intermediate haplotype diversity between the African and European samples, and the Mexican Mestizo sample has intermediate haplotype diversity between the European and Native American samples (Figure S4).

In Table 2, we show the frequencies of alleles/haplotypes of PGx relevance for the *CYP2D6*, *UGT1A1* and *VKORC1* genes, which can be inferred based on the markers of the DMET Plus array. Although, unfortunately the array does not capture all the functional variants described for these genes, such as UGT1A1*28. The allele frequencies of several *CYP2D6* variants of functional importance are known to vary amongst ethnic groups, and this explains variation in interindividual drug response [30], [31]. The frequencies of the non-functional CYP2D6*4 allele, and the decreased-activity allele CYP2D6*41 are considerably higher in Europeans than in Native American or West African populations. As expected, the relative frequencies for these alleles are intermediate in the admixed samples, and the variants are present in frequencies that are proportional to the relative admixture proportions. In the case of *UGT1A1*, the frequencies of variants that have been associated with irinotecan response *(−3156G>A/−349C>T* and UGT1A1*60) [32], [33], [34], [35] are also higher in European than Native American and West African populations. The consistent higher frequencies of the functional *CYP2D6* and *UGT1A1* alleles/haplotypes observed in Europeans vs. the other two populations are probably the result of ascertainment bias, because most of the studies exploring variants with potentially functional effects have been carried out in European populations. Finally, for *VKORC1* there are also clear differences across populations in the distribution of haplotypes associated with low (H1 and H2) and high (H7/H8 and H9) warfarin dose requirements [36]. Again, in the admixed samples the frequencies follow a gradient depending on admixture proportions.

**Table 2:** Frequencies of alleles/haplotypes in CYP2D6, UGT1A1 and VKORC1 genes in Mexicans and Brazilians populations.

Gene	Allele/haplotype	Frequency							Functional effect
		EUR	Brazilian White	Brazilian Brown	Brazilian Black	AFR	MEX	NAT	
	*1	0.47	0.34	0.42	0.37	0.31	0.59	0.88	Normal enzyme activity
CYP2D6^1^^,^ 2	*2	0.21	0.28	0.21	0.13	0.05	0.21	0.02	Normal enzyme activity
	*4	0.24	0.21	0.16	0.09	0.07	0.14	0.10	Non-functional allele
	*41	0.08	0.08	0.02	0.01	0.02	0.03	0.00	Decreased function allele
	*1	0.44	0.32	0.27	0.18	0.06	0.26	0.19	Normal enzyme activity
UGT1A1^3^	−3156G>A/−349C>T	0.23	0.22	0.21	0.11	0.08	0.11	0.03	Toxicity in irinotecan treatment
	*60	0.05	0.05	0.04	0.01	0.01	0.02	0.00	Toxicity in irinotecan treatment
	H1	0.11	0.05	0.08	0.09	0.04	0.06	0.00	Low-dose warfarin
VKORC1^4^	H2	0.26	0.24	0.23	0.08	0.00	0.10	0.02	Low-dose warfarin
	Set H7/H8	0.37	0.30	0.24	0.28	0.25	0.43	0.48	High-dose warfarin
	H9	0.25	0.21	0.15	0.11	0.04	0.08	0.00	High-dose warfarin
								

1 The Human Cytochrome P450 (*CYP*) Allele Nomenclature Database Website. Available: http://www.cypalleles.ki.se/cyp2d6.htm. Accessed 2013 November 14.

2 Lyon E,. et al. (2012) Genet Med 14(12): 990–1000.

3 Whirl-Carrillo M. et al. (2012) Clin Pharmacol Ther 92(4): 414–7.

4 Reference number [36].

### Identification of DMET Plus markers showing extreme genetic differentiation

We identified the genetic markers included in the DMET Plus array that show the most extreme genetic differentiation (e.g. highest *F_ST_* values) between the parental populations that are most relevant for the Brazilian (European and African populations) and Mexican Mestizo (European and Native American) samples. Tables 3 and 4 list the 18 SNPs with the most extreme genetic differentiation between Europeans and either Africans or Native Americans, respectively. All these SNPs have a drug association reported in the Pharmacogenomics Knowledge Database (PharmGKb, www.pharmgkb.org), and their *Bonferroni* adjusted p-values are less than 1×10^−20^ in the European-African comparison and less than 1×10^−6^ in the European-Native American comparison. The SNPs listed in Tables 3 and 4 are located in genes belonging to the Cytochrome P450, Solute Carrier (SLC) and UDP-glucuronyltransferase (UGT) families as well as other genes of PGx relevance such as *ABCC8, ADH1A, CHST3, PON1, PPARD, PPARG,* and *VKORC1*. Of note, 6 of the SNPs identified in the African-European comparison are also present in the European-Native American comparison: rs2470890 at *CYP1A2*, rs2242480 at *CYP3A4*, rs1050152 at *SLC22A4*, rs2242046 at *SLC28A1*, rs1060896 at *SLC28A2* and rs7867504 at *SLC28A3*. The list of markers with high genetic differentiaton for Brazilians and Mexicans is presented in Tables S3 and S4. All frequencies data can be consulted in Table S5.

**Table 3:** Distribution of most frequent pharmacogenetic polymorphisms in Europeans compared with Africans.

SNP	Gene	Allele	EUR	AFR		
			MAF	Freq	Fst φ	p-value*
rs776746	CYP3A5	T	0.06	0.86	0.639	1.73E-57
rs2242480 Δ	CYP3A4	T	0.08	0.87	0.611	5.41E-55
rs2740574	CYP3A4	C	0.03	0.79	0.609	1.79E-53
rs1402467 Δ	SULT1C2	G	0.19	0.95	0.590	1.17E-58
rs2470890	CYP1A2	C	0.35	1	0.484	6.97E-60
rs6922548	PPARD	G	0.08	0.76	0.476	1.01E-39
rs1060896 Δ	SLC28A2	A	0.73	0.10	0.409	1.76E-37
rs2070673 Δ	CYP2E1	A	0.17	0.80	0.394	8.63E-33
rs2515641	CYP2E1	T	0.10	0.68	0.354	5.93E-28
rs7867504 Δ	SLC28A3	C	0.33	0.87	0.312	2.14E-27
rs1050152 Δ	SLC22A4	T	0.46	0.00	0.304	6.69E-39
rs7311358	SLCO1B3	G	0.14	0.67	0.297	6.95E-23
rs4149117	SLCO1B3	T	0.14	0.67	0.295	9.62E-23
rs2242046 Δ	SLC28A1	A	0.51	0.02	0.290	1.41E-31
rs4148945 Δ	CHST3	C	0.47	0.94	0.268	1.05E-25
rs17878544	VKORC1	C	0	0.40	0.253	8.07E-20
rs4124874 Δ	UGT1A1	G	0.42	0.90	0.252	2.45E-22
rs757110 Δ	ABCC8	C	0.39	0.00	0.237	1.27E-29

* p-value is for the comparison of the allele frequencies for EUR and other populations; adjusted p-value by Bonferroni correction.

φ *Fst* for SNP based on allele frequencies in Ancestral populations.

Δ SNP is an AIM.

**Table 4:** Distribution of most frequent pharmacogenetic polymorphisms in Europeans compared with Native Americans.

SNP	Gene	Allele	EUR	NAT		
			MAF	Freq	Fst φ	p-value*
rs1060896 Δ	SLC28A2	C	0.27	0.98	0.552	7.91E-27
rs2470890	CYP1A2	C	0.35	1	0.484	8.32E-24
rs2069514	CYP1A2	A	0.02	0.58	0.376	1.20E-18
rs28365062	UGT2B7	G	0.18	0.81	0.401	1.87E-17
rs2242046 Δ	SLC28A1	A	0.51	0.00	0.340	3.76E-16
rs1048943	CYP1A1	C	0.04	0.58	0.335	1.52E-15
rs11150606 Δ	VKORC1	C	0.02	0.50	0.304	9.75E-15
rs2242480 Δ	CYP3A4	T	0.08	0.63	0.327	2.30E-14
rs1050152 Δ	SLC22A4	T	0.47	0.01	0.285	1.21E-12
rs6811453	ADH1A	A	0.42	0.00	0.268	2.7E-12
rs1826909	ADH1A	T	0.41	0.00	0.255	1.58E-11
rs7867504 Δ	SLC28A3	C	0.33	0.84	0.272	9.62E-11
rs4646	CYP19A1	A	0.22	0.73	0.264	1.44E-10
rs4803418	CYP2B6	G	0.29	0.81	0.267	0.00
rs854560	PON1	T	0.42	0.02	0.226	3.01E-09
rs1152003	PPARG	G	0.31	0.79	0.230	1.18E-08
rs7662029 Δ	UGT2B7	A	0.49	0.09	0.190	0.000000363
rs628031 Δ	SLC22A1	A	0.39	0.04	0.175	1.74E-06

*p-value is for the comparison of the allele frequencies for EUR and other populations; adjusted p-value by Bonferroni correction.

φ *Fst* for SNP based on allele frequencies in Ancestral populations.

Δ SNP is an AIM.

**Figure 2:**
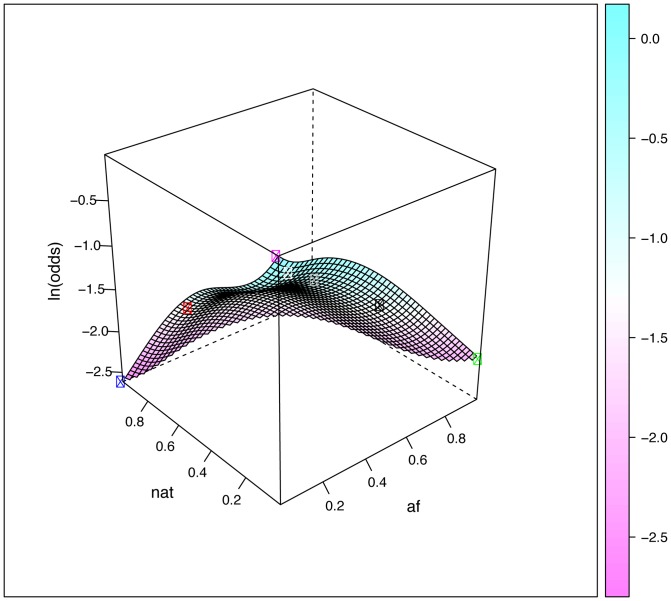
Surface plots describing the predicted relationship between the distribution of rs2470890 and parental ancestry, obtained by fitting piece-wise smooth logistic regression models to the 268 Brazilians and 224 Mexicans data. The relative proportions of Native American and African ancestry are plotted in the x and y axes as appropriately labeled, whereas the relative proportion of European ancestry is inferred as the remaining proportion: 1- (af+nat). The plotted surface corresponds to the natural logarithm of the odds of having the variant rs2470890 T allele, depending on the relative admixture proportions of the parental populations. The odds refer to the ratio of the variant:wild-type alleles. For example the odds of having a variant allele frequency of 0.1 (wild-type frequency = 0.9) is 01/09 or 0.111. The corresponding Ln odds is −2.197. The circles correspond to the average ancestral proportions for Black Brazilians (black circle), Brown Braziilans (grey), White Brazilians (white), Mexicans (red), EUR (yellow), AFR (green) and NAT (blue).

We applied non-linear piece-wise smooth logistic regression modeling (see Materials and methods section) to explore how individual ancestry proportions influence allele frequencies for *CYP1A2* rs2470890, which is one of the six SNPs that showed extreme genetic divergence between Europeans and both Africans and Native Americans. The resulting three-dimensional plot is presented in Figure 2.

### Impact of admixture history at the population level on PGx implementation

**Figure 3:**
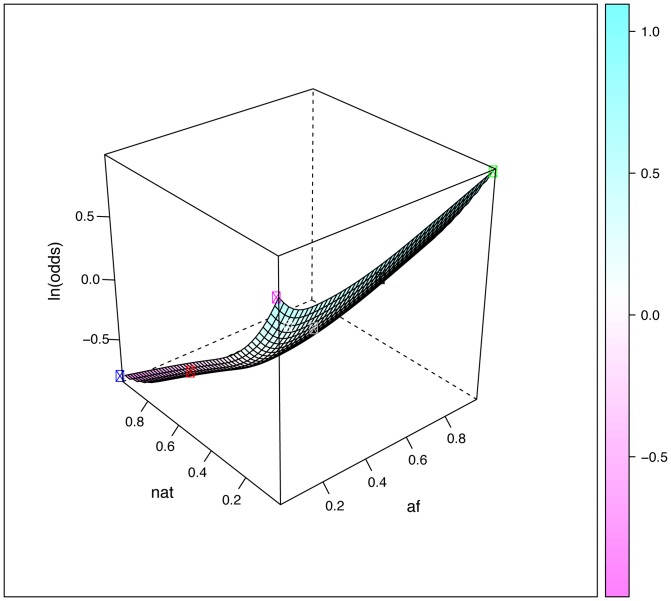
Surface plots describing the predicted relationship between the frequency of rs1135840 and parental ancestry, obtained by fitting piece-wise smooth logistic regression models to the 268 Brazilians and 224 Mexicans data. The relative proportions of Native American, African ancestry and European ancestry are presented as described in Figure 2. The plotted surfaces correspond to the natural logarithm of the odds of having the variant rs1135840 G allele, depending on the relative admixture proportions of the parental populations. The circles correspond to the average ancestral proportions for Black Brazilians (black circle), Brown Braziilans (grey), White Brazilians (white), Mexicans (red), EUR (yellow), AFR (green) and NAT (blue).

**Figure 4:**
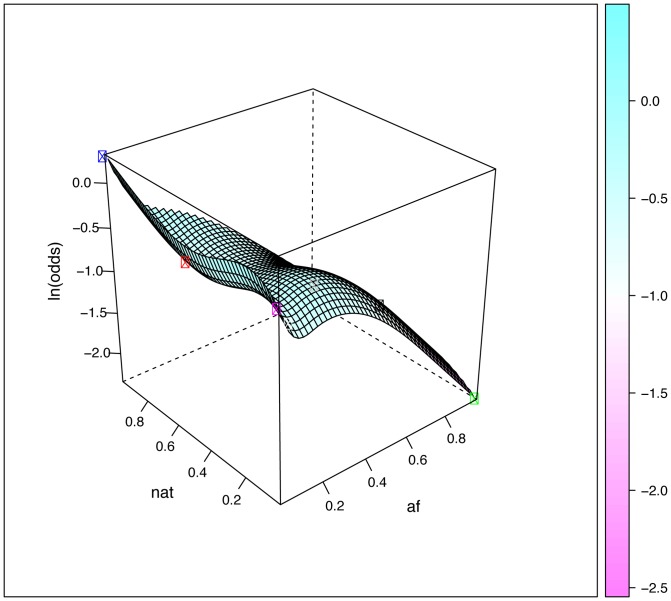
Surface plots describing the predicted relationship between the frequency of rs9923231 and parental ancestry, obtained by fitting piece-wise smooth logistic regression models to the 268 Brazilians and 224 Mexicans data. The relative proportions of Native American, African and European ancestry are presented as described in Figure 2. The plotted surfaces correspond to the natural logarithm of the odds of having the variant rs9923231 G allele, depending on the relative admixture proportions of the parental populations. The circles correspond to the average ancestral proportions for Black Brazilians (black circle), Brown Brazilians (grey), White Brazilians (white), Mexicans (red), EUR (yellow), AFR (green) and NAT (blue).

Eighteen SNPs in the DMET array are included in the published guidelines of the Clinical Pharmacogenetics Implementation Consortium (CPIC, www.pharmgkb.org/page/cpic). We selected these SNPs to examine the impact of admixture on the distribution of clinically-important PGx polymorphisms in Brazilians and Mexicans. Initially, we calculated the *F_ST_* values for each locus in pair-wise comparisons of proxy parental populations, namely EUR *versus* AFR (most relevant for Brazilians) and EUR *versus* NAT (most relevant for Mexicans). The average *F_ST_* for the 18 markers was 0.037 in EUR *versus* AFR and 0.044 in EUR *versus* NAT (Table 5). Similarly to what was found for all the markers surveyed by the DMET Plus array (see above), these *F_ST_* values point to low genetic differentiation. However, some SNPs displayed substantially higher levels of genetic differentiation (Table 5). Thus, 4 markers in the EUR - AFR comparison and 3 markers in the EUR - NAT comparison showed moderate divergence (*F_ST_*>0.05), whereas large genetic divergence (*F_ST_*>0.15) was observed for rs1135840 in *CYP2D6* between EUR and NAT, and for rs9923231 in *VKORC1* between EUR and AFR. We show the three-dimensional plots for these two markers in Figures 3 and 4, respectively. Figure S5 shows a three-dimensional plot for a marker, rs16947 in *CYP2D6,* which shows moderate genetic divergence between EUR and both AFR and NAT.

**Table 5:** *F*_ST_ values for pair-wise comparisons between the HapMap EUR and AFR groups and between EUR and NAT.

Gene	rs ID	*F_ST_*	
		EUR:AFR	EUR:NAT
*CYP2C19*	rs12248560	0	0.124
*CYP2C19*	rs4244285	0.003	0.049
*CYP2C19*	rs4986893	0.001	0
*CYP2C9*	rs1057910	0.031	0.031
*CYP2C9*	rs1799853	0.083	0.083
*CYP2D6*	rs1065852	0.050	0.034
*CYP2D6*	rs1135840	0.026	0.192
*CYP2D6*	rs16947	0.102	0.140
*CYP2D6*	rs28371725	0.022	0.044
*CYP2D6*	rs3892097	0.080	0.034
*DPYD*	rs3918290	0	0
*DYPD*	rs55886062	0	0
*SLCO1B1*	rs4149056	0.070	0.037
*TPMT*	rs1142345	0.017	0.006
*TPMT*	rs1800460	0.013	0.006
*TPMT*	rs1800462	0	0
*TPMT*	rs1800584	0	0
*VKORC1*	rs9923231	0.174	0.020
**mean**		**0.037**	**0.044**

## Discussion

Here, we describe the distribution of genetic markers of PGx relevance in two samples from the two most populous countries in Latin America, Brazil and Mexico. The Brazilian sample comes from the State of Rio de Janeiro, in Southeast Brazil, and was stratified according to the census categories used in this country. The Mexican sample comprised individuals from an indigenous population, the Zapotecas, and also Mestizo individuals from five States located in different regions of Mexico.

We evaluated the distribution of *F_ST_* values of the genetic markers included in the DMET Plus array between pairs of ancestral populations. The *F_ST_* statistic provides an indication of the level of genetic differentiation between populations. The distribution of *F_ST_* values of the DMET Plus markers mirrors the *F_ST_* distribution that has been described for much denser panels of markers [37], [38]. Overall, the amount of genetic differentiation is low, but there is a long tail of markers showing substantial genetic differentiation (Figure S3). The highest average levels of genetic differentiation were found between the African and Native American samples (average *F_ST_*  = 0.085). The estimates of genetic (haplotype) diversity based on the DMET Plus data are also consistent with evidence based on dense genome-wide data [39], [40]. The highest haplotype diversity is found in the African sample and the lowest in the Native American sample. These differences in genetic diversity have been attributed to serial founder effects after the migration of modern humans out of Africa [41], [42]. As expected, the haplotype diversities in the admixed samples from Brazil and Mexico are consistent with the known history of admixture of each region. The Brazilian samples have intermediate levels of diversity between the African and European samples, and the Mexican samples have intermediate levels of diversity between the Native American and European samples (Figure S4).

The admixture proportions of the individuals of Brazil and Mexico were estimated using a panel of highly informative AIMs included in the array. We selected 71 unlinked markers based on the degree of genetic differentiation between the relevant ancestral populations. The number of AIMs was limited due to the relatively low number of markers included in the DMET array (approximately 2,000) and the fact that many of the markers are in linkage disequilibrium and do not provide independent information. However, the ancestry information content of the panel is high and the individual admixture estimates obtained with the DMET AIMs panel are highly correlated with admixture estimates based on genome-wide data (Affy 100K and Illumina 550K) in a subset of the Mexican samples (see Results section above). The estimates of the average proportions of European, African and Native American ancestry in the Brazilian sample from Rio de Janeiro are very similar to those reported by Pena et al. [10] using an independent set of AIMs for a different cohort of White, Brown and Black Brazilians from the same geographical region.

Two issues must be highlighted regarding the results of the admixture analysis. First of all, there is a broad distribution of individual ancestry proportions in the samples from Brazil and Mexico (Figure 1 and Figure S1). Secondly, there are substantial differences in admixture proportions between census groups in Brazil and geographic regions in Mexico (Table 1). In Brazil, the average contribution of European ancestry decreased progressively from self-reported White (84.6%), to Brown (64.7%) and then to Black individuals (38.1%), whereas the opposite trend was observed with respect to African ancestry, which averaged 9.7%, 25% and 54% in White, Brown and Black persons, respectively. Native ancestry ranged from 5.6 to 10% across the three groups. The Brazilian individuals included in this study came exclusively from Rio de Janeiro (Southeast Brazil), so it was not possible to evaluate geographic variation in admixture proportions in Brazil. However, there is evidence pointing to the presence of substantial variation in admixture proportions in different regions of Brazil, even within census categories. For instance, [10] recently reported that self-classified Brown individuals from the North region had on average 68.6% European ancestry, compared to 44.4% in the South region. Similarly, European ancestry in self-reported Blacks ranged from 29.3% in the South to 53.9% in the Northeast region. This variability reflects the fact that self-identification based on the “race/Color” categorizations for Brazilians in our manuscript correspond to the categories adopted by the official Brazilian Census, where White, Brown and Black categories for Brazilians is influenced by phenotypic variables such as skin and eye pigmentation and facial features, as well as family history, sunlight exposure, income level, social class and schooling [10], [42], [43], [44]. Collectively, these factors underlie the tenuous correlation between self-reported color and biogeographical ancestry among Brazilian [10], [46].

**Figure 5:**
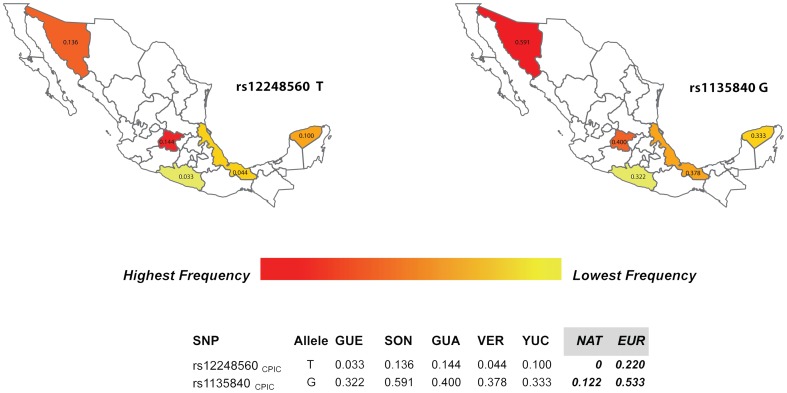
Frequency distributions of DMET Plus markers by region within Mexico. We analyzed the distribution of the DMET Plus markers by region. CPIC marker rs12248560 shows much lower frequency for those states with stronger Native American component (GUE, VER) almost 4-fold when compared to that of SON. Whilst, rs1135840 shows that frequency for the state of SON is almost 2-fold when compared to that of GUE, a state with a high Native American component. This also correspond to what is observed at the ancestral population levels, over 4-fold difference between European and Native American.

Based on the results obtained with the panel of AIMs, in Mexico there is also evidence of regional variation in admixture proportions, in particular between the northern State of Sonora and the other States. The European admixture proportions in Sonora are 50% higher than the proportions observed in the Southern State of Guerrero. This geographic variation in admixture proportions within Mexico has also been described based on genome-wide panels of markers [11]. Indeed, when we analyzed the distribution of the DMET Plus markers by region within Mexico, we observed that a number of markers show substantial variation between regions, including a marker of clinical relevance (rs12248560) located in the *CYP2C19* gene, which has been classified by CPIC as CPIC level A (annotation used to indicate that genetic information should be used to change prescribing of affected drug) and PharmGKB level 1A (annotation used for variant-drug combinations in a CPIC or medical society-endorsed PGx guideline). The *CYP2C19* allele rs12248560 T (CYP2C19*17) is an ultrarapid metabolizer of drugs such as amitriptyline and clopidogrel. The frequency of this allele is more than 4-fold higher in states with high European admixture proportions (e.g Sonora: 0.144 and GUA: 0.136) than in states with high Native American admixture proportions (e.g. GUE: 0.033) (Figure 5).

As stated above, although the overall genetic differentiation of the markers interrogated in the DMET Plus array is low, there are many loci that show high levels of genetic differentiation between the parental populations relevant for contemporary Brazilians and Mexicans. Given the broad range of admixture proportions observed in Brazil (both in terms of census categories and geographic regions) and Mexico (geographic regions), this has clear PGx implications. We displayed graphically in Figure 2, 3 and 4 the impact of admixture proportions using three-dimensional graphs that depict the relationship of alleles and ancestry. The odds of having a variant of PGx relevance vary over ranges determined by the frequency of the polymorphisms in the relevant ancestral populations, i.e. mainly Europeans and Africans for Brazilians, and European and Native Americans for Mexicans. The larger the difference in frequency between the relevant parental populations, the more inappropriate it is to refer to a “Brazilian” or “Mexican” allele frequency. In reality, there will be variation in allele (and genotype) frequencies depending on geographic areas or census categories. Therefore, it may be misleading to extrapolate the allele (and genotype) frequencies found in a given geographic area or census group to another geographic area or census group. In addition, most of the available data are from people of European ancestry, so clinical translation in non-Europeans can be challenging [45], thus our results highlight the importance of studies in admixed populations because they present opportunities for discovery of genetic markers that would be missed.

The *VKORC1* rs9923231 SNP, a major determinant of warfarin dose in CPIC guidelines, may be used as an example of the practical PGx implications of admixture history. The frequency of the rs9923231T allele, which associates with high warfarin sensitivity, varies 13-fold among proxy parental populations of Brazilians and Mexicans: 4.1% in sub-Saharan Africans (HapMap YRI and LWK), 38.1% in Europeans (HapMap CEU) and 52.2% in Native Americans (Zapotecas). Limdi et al [4] showed that the proportion of variation in warfarin dose explained by the *VKORC1* rs9923231 SNP increases as the frequency of the A allele increased, such that *VKORC1* explained greater variability in dose among Europeans compared to Africans and African Americans. Accordingly, warfarin-dosing algorithms including *VKORC1* rs9923231 as a co-variate have considerably greater predictive power in Europeans compared to Africans [1]. However, the predictive power of two such algorithms did not differ between White and Black Brazilians: this was explained by the higher frequency of the rs9923231T allele in Black Brazilians, as a result of the extensive European- African admixture [5], [47].

## Conclusions

In summary, our study emphasizes the remarkable population complexity found in Brazil and Mexico: there is a broad range of admixture proportions within census categories and geographic regions, which is a reflection of differences in population history. It is important to consider that, although Brazil and Mexico are the largest countries in Latin America, they only represent a subset of the diversity observed in this vast geographic area. It is therefore critical to have this rich diversity in mind when considering the PGx impact of admixture [45], [48]. In this sense, estimating admixture proportions at the population or individual level can be useful in two respects. At the population level, knowing average admixture proportions it is possible to infer the allele (and genotype) frequencies of relevant PGx variants. In turn, this distribution determines the proportion of the variance of traits of PGx importance explained by the polymorphisms. At the individual level, individual ancestry proportions will determine the probability of having a PGx relevant genotype. For these reasons, it is important to carry out further efforts to characterize admixture in the Americas. In particular, our understanding of the variation in admixture proportions within countries is still quite incomplete, and there are many gaps in our knowledge of the frequency distributions in the relevant parental populations (in particular, Native American groups).

## Supporting Information

Figure S1 Population structure analysis using 71 AIMs. Individual ancestry proportions in Brazilians (BR.BK, BR.BN, BR.WT) and Mexican Mestizo (MEX).
(TIF)

Figure S2 Analysis of correlation of individual ancestry estimates for Mexican Mestizo individuals for which data are available for the DMET Plus array and genome-wide arrays. Data for 74 individuals were available for both the DMET Plus array and the Illumina 550K array. Data for 68 individuals were available for both the DMET Plus array and the Affymetrix 100K array. Individual ancestry estimates for the Illumina 550K array were obtained with the program ADMIXTURE. Individual ancestry estimates for the Affymetrix 100K array were obtained with the program STRUCTURE using 1814 AIMs [11].
(TIF)

Figure S3 Distribution of *F_ST_* values. Pairwise comparisons were done for the three ancestral populations and for the admixed populations Mexico and Brazil, a) Europe vs Africa; b) Europe vs Native American; c) Africa vs Native American; and d) Mexico vs Brazil.
(TIF)

Figure S4 Percentage of total common haplotypes observed per population. In order to prepare this plot, first we identified all the haplotypes present in each sample with a frequency equal or higher than 5%. The combined number of haplotypes observed was 1017. The Bar plots represent the percentage of haplotypes observed in each individual sample (e.g. 100% would correspond to the 1017 haplotypes observed in the combined sample). The data set presented includes all the Mexican admixed samples (MEX(MX)), all the Brazilian census groups (BZ.BK, BZ.BN, BZ.WT), the Native American Zapoteca sample (NAT(ZAP)), and several HapMap samples (EUR, AFR, MEX(LA), JPT.CHB).
(TIF)

Figure S5 Surface plots describing the predicted relationship between the frequency of rs16947 and parental ancestry, obtained by fitting piece-wise smooth logistic regression models to the 268 Brazilians and 224 Mexicans data. The relative proportions of Native American, African ancestry and European ancestry are presented as described in Figure 2. The plotted surfaces correspond to the natural logarithm of the odds of having the variant rs16947A allele, depending on the relative admixture proportions of the parental populations. The circles correspond to the average ancestral proportions for Black Brazilians (black circle), brown Braziilans (grey), White Brazilians (white), Mexicans (red), EUR (yellow), AFR (green) and NAT (blue).
(TIF)

Table S1 Allele Frequencies and FST values for 71 Ancestry Informative Markers (AIMs) in ancestral populations.
(XLSX)

Table S2 FST values for all pairwise comparisons among HapMap populations, Zapotecos (NAT), Brazilians (BK, BN, WT) and Mexicans (MEX).
(XLSX)

Table S3 Polymorphisms showing the highest genetic differentiation between Native Americans and the Admixed populations.
(XLSX)

Table S4 Polymorphisms showing the highest genetic differentiation between Africans and the Admixed populations.
(XLSX)

Table S5 Minor Allele Frequencies (MAF) for 1,647 genetic markers. The markers are those included in the DMET Plus array and for all populations: Europeans (Hapmap CEU), Africans (Hapmap YRI and LWK), Native Americans (Zapotecas), Brazilians and Mexican mestizos (Guanajuato (GUA); Guerrero (GUE); Sonora (SON); Veracruz (VER) and Yucatan (YUC)).
(XLSX)

Data S1 Genotype data with 1,647 genetic markers from 537 individuals form Brazil and Mexico.
(ZIP)
