# Correction: Exploring the Distribution of Genetic Markers of Pharmacogenomics Relevance in Brazilian and Mexican Populations

**DOI:** 10.1371/journal.pone.0122161

**Published:** 2015-03-23

**Authors:** The PLOS ONE Staff

There is an error in the second sentence in the third paragraph of the “Genetic differentiation and haplotype diversity for DMET Plus markers” section of the Results. The correct sentence is: Although, the array captures many of the functional variants described for these genes, unfortunately UGT1A1*28 was not questioned in our study.

The S5 Table file does not open properly. Please view the correct S5 Table below.

## Supporting Information

S5 Table Supplementary dataset. Minor Allele Frequencies (MAF) for 1,647 genetic markers. The markers are those included in the DMET Plus array and for all populations: Europeans (Hapmap CEU), Africans (Hapmap YRI and LWK), Native Americans (Zapotecas), Brazilians and Mexican mestizos (Guanajuato (GUA); Guerrero (GUE); Sonora (SON); Veracruz (VER) and Yucatan (YUC)).
(XLSX)
