# Supplementary figures and images for: Exploring the Distribution of Genetic Markers of Pharmacogenomics Relevance in Brazilian and Mexican Populations
Source: PLoS One. 2014 Nov 24;9(11):e112640. doi: 10.1371/journal.pone.0112640 (PMC4242606; doi:10.1371/journal.pone.0112640)

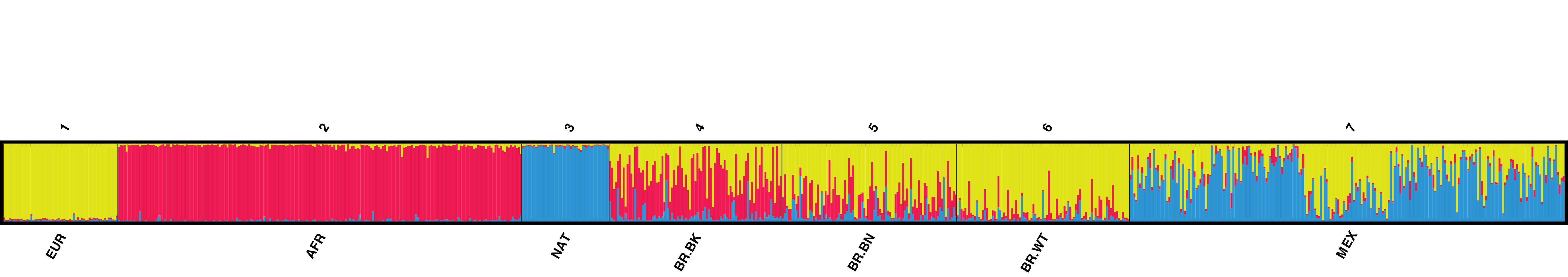

Supplement: Figure S1 — Population structure analysis using 71 AIMs. Individual ancestry proportions in Brazilians (BR.BK, BR.BN, BR.WT) and Mexican Mestizo (MEX). (TIF) [file pone.0112640.s001.tif]

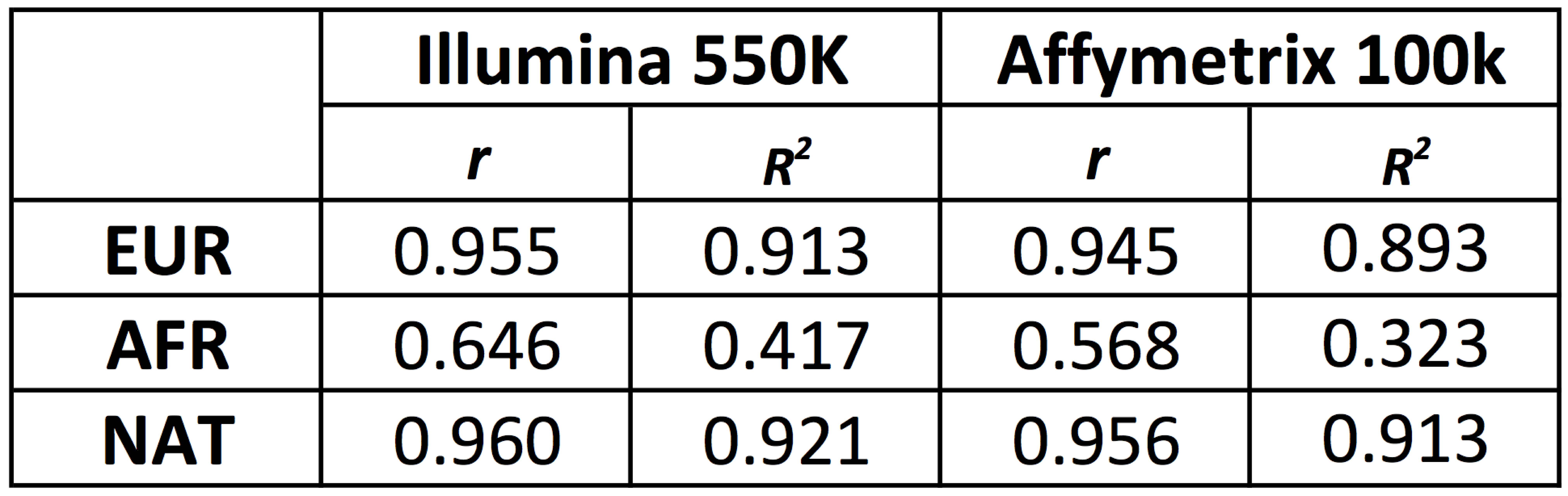

Supplement: Figure S2 — Analysis of correlation of individual ancestry estimates for Mexican Mestizo individuals for which data are available for the DMET Plus array and genome-wide arrays. Data for 74 individuals were available for both the DMET Plus array and the Illumina 550K array. Data for 68 individuals were available for both the DMET Plus array and the Affymetrix 100K array. Individual ancestry estimates for the Illumina 550K array were obtained with the program ADMIXTURE. Individual ancestry estimates for the Affymetrix 100K array were obtained with the program STRUCTURE using 1814 AIMs [11]. (TIF) [file pone.0112640.s002.tif]

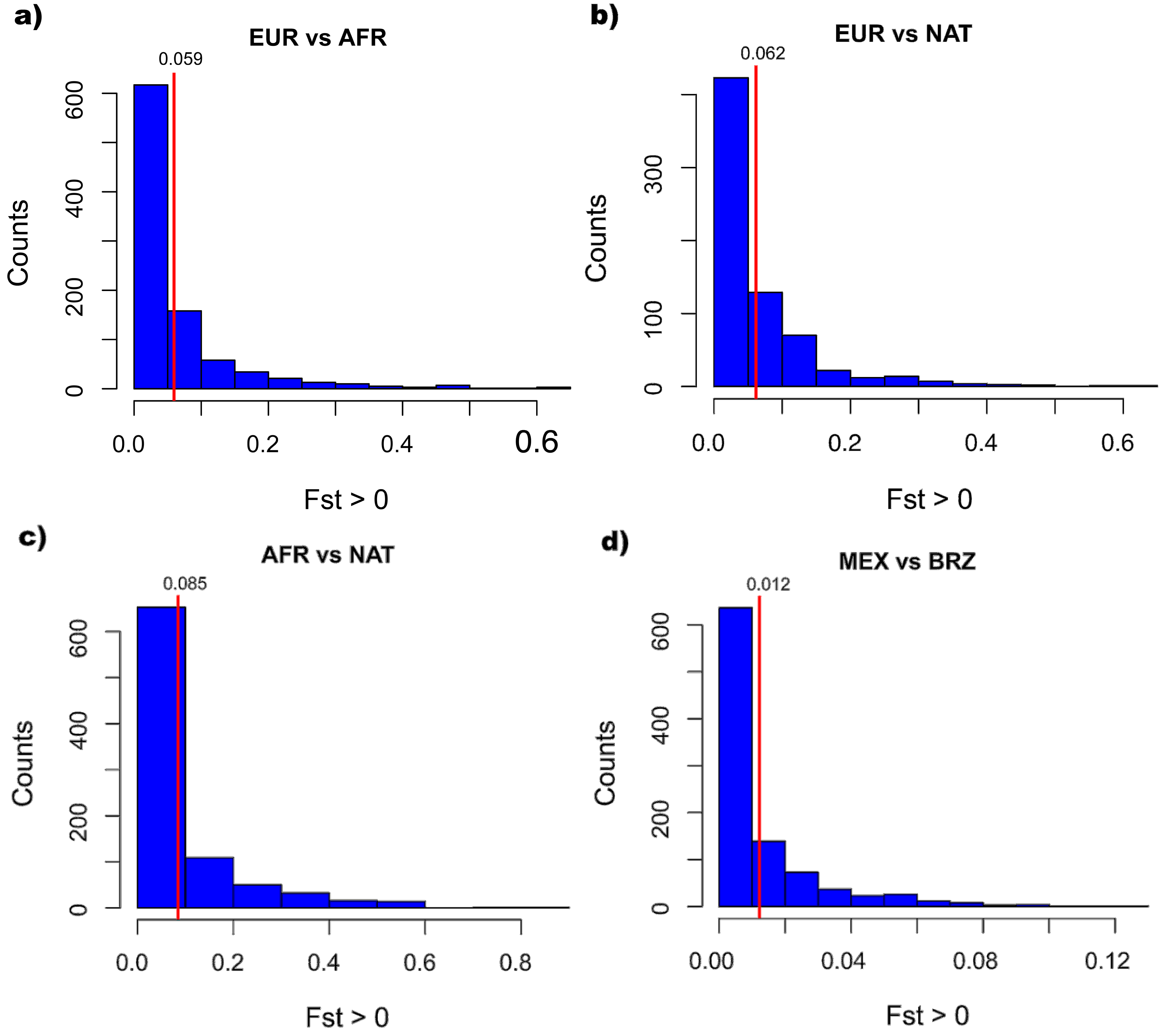

Supplement: Figure S3 — Distribution of FST values. Pairwise comparisons were done for the three ancestral populations and for the admixed populations Mexico and Brazil, a) Europe vs Africa; b) Europe vs Native American; c) Africa vs Native American; and d) Mexico vs Brazil. (TIF) [file pone.0112640.s003.tif]

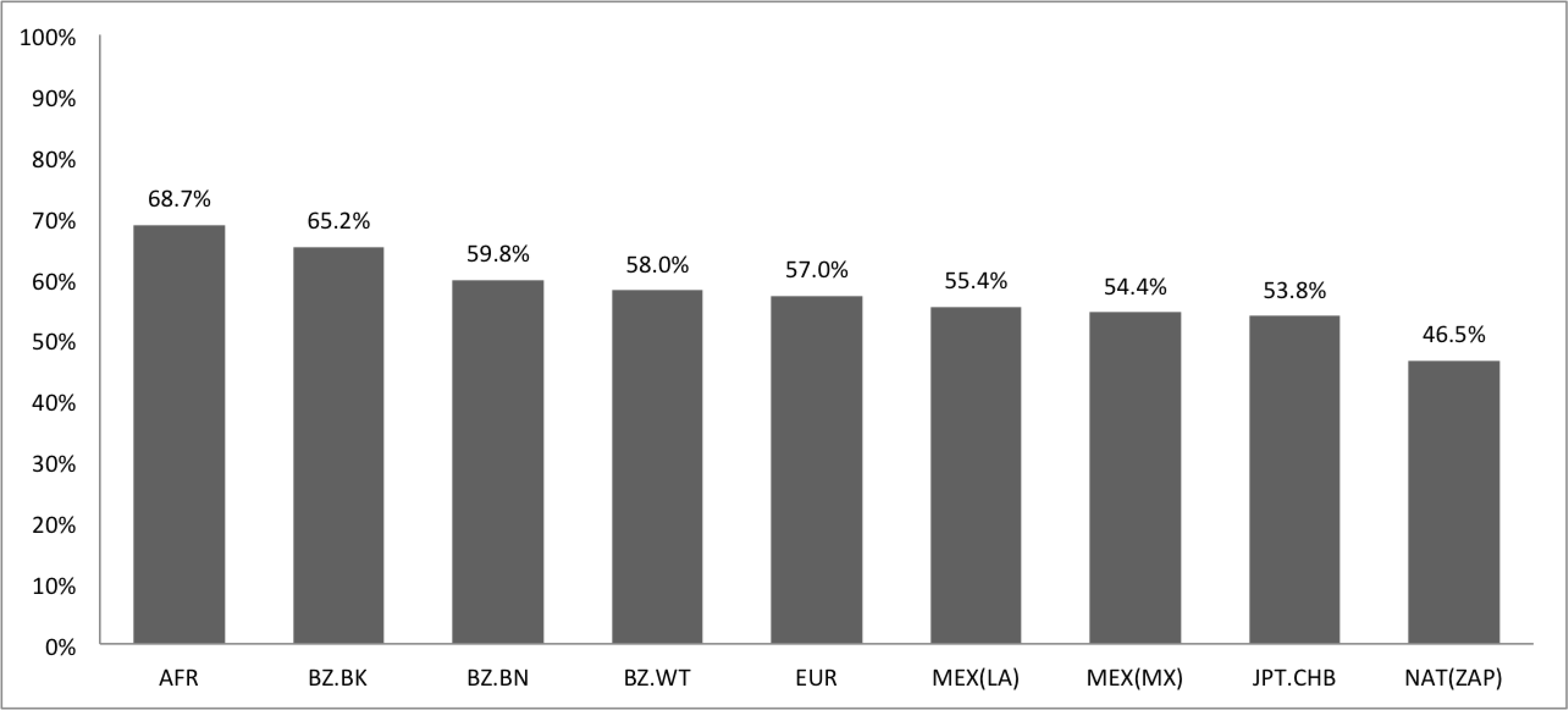

Supplement: Figure S4 — Percentage of total common haplotypes observed per population. In order to prepare this plot, first we identified all the haplotypes present in each sample with a frequency equal or higher than 5%. The combined number of haplotypes observed was 1017. The Bar plots represent the percentage of haplotypes observed in each individual sample (e.g. 100% would correspond to the 1017 haplotypes observed in the combined sample). The data set presented includes all the Mexican admixed samples (MEX(MX)), all the Brazilian census groups (BZ.BK, BZ.BN, BZ.WT), the Native American Zapoteca sample (NAT(ZAP)), and several HapMap samples (EUR, AFR, MEX(LA), JPT.CHB). (TIF) [file pone.0112640.s004.tif]

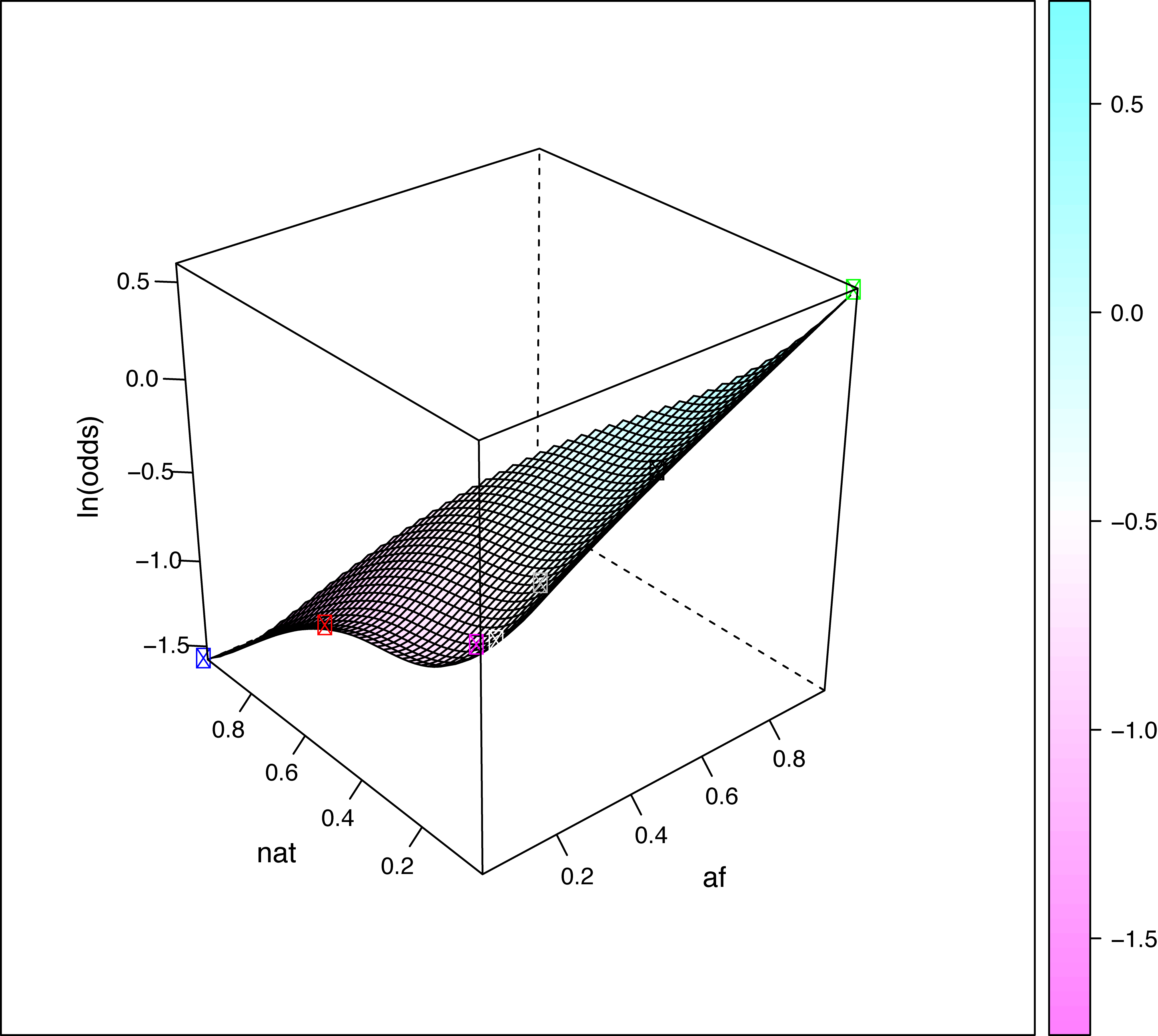

Supplement: Figure S5 — Surface plots describing the predicted relationship between the frequency of rs16947 and parental ancestry, obtained by fitting piece-wise smooth logistic regression models to the 268 Brazilians and 224 Mexicans data. The relative proportions of Native American, African ancestry and European ancestry are presented as described in Figure 2. The plotted surfaces correspond to the natural logarithm of the odds of having the variant rs16947A allele, depending on the relative admixture proportions of the parental populations. The circles correspond to the average ancestral proportions for Black Brazilians (black circle), brown Braziilans (grey), White Brazilians (white), Mexicans (red), EUR (yellow), AFR (green) and NAT (blue). (TIF) [file pone.0112640.s005.tif]
